# Health-Related Internet Use by Informal Caregivers of Children and Adolescents: An Integrative Literature Review

**DOI:** 10.2196/jmir.4124

**Published:** 2016-03-03

**Authors:** Eunhee Park, Heejung Kim, Andreanna Steinhoff

**Affiliations:** ^1^ University of North Carolina at Greensboro School of Nursing Greensboro, NC United States; ^2^ Yonsei University College of Nursing Seoul Republic Of Korea; ^3^ University of Kansas Medical Center School of Nursing University of Kansas Kansas City, KS United States

**Keywords:** Internet, caregivers, children, adolescent, eHealth, consumer health information

## Abstract

**Background:**

Internet-based health resources can support informal caregivers who are caring for children or adolescents with health care needs. However, few studies discriminate informal caregivers’ needs from those of their care recipients or those of people caring for adults.

**Objective:**

This study reviews the literature of health-related Internet use among informal caregivers of children and adolescents.

**Methods:**

A total of 17 studies were selected from literature searches conducted in 6 electronic databases: PubMed, Cochrane, CINAHL, PsycINFO, ERIC, and EMBASE. All databases searches were limited to articles published in the years 2004 to 2014 in peer-reviewed publications. Search terms consisted of “health-related Internet use,” “eHealth,” “Internet use for health-related purpose(s),” “Web-based resource(s),” and “online resources,” combined with informal caregiver (or “parents”) of “child,” “adolescent,” “student,” “youth,” and “teen.” The age range of the children receiving care was limited to younger than 22 years. Their informal caregivers were defined as persons (parents) who provided unpaid care or assistance to a child or an adolescent with health problems.

**Results:**

Among 17 empirical studies, the majority of informal caregivers of children with medical issues were the parents. Quantitative studies (14/17, 77%) reported prevalence and predictors of health-related Internet use, while mixed-methods and qualitative studies (3/17, 24%) investigated informal caregiver perceptions of helpful health-related Internet use and barriers of use. The prevalence of health-related Internet use varied (11%-90%) dependent upon how health-related Internet use was operationalized and measured. Disease-specific information was used for decision making about treatment, while social support via virtual communities and email were used for informal caregiver emotional needs. A digital divide of Internet access was identified in lower educated minorities. Most studies had methodological challenges resulting from convenience sampling, cross-sectional surveys, lack of theoretical frameworks, or no clear definitions of health-related Internet use.

**Conclusions:**

This study provides an important understanding of how family members use Internet-based information and support systems during child caregiving. Healthcare providers and policy makers should integrate family needs into their current practices and policies. Further rigorous research is required to design efficient and effective nursing interventions.

## Introduction

The Internet continues to play an increasingly important role in our everyday lives, particularly regarding the delivery of health care services and interventions. Health-related Internet use is defined as any activity involving Internet-based information and resources for improving health and well-being [1-3]. Characteristics of the Internet that are important in delivering health care services and resources include: (1) medical information and health care resources can be accessed from diverse locations; (2) interactive features allow people to be more proactive health care consumers; and (3) Internet-based health resources can support patients and their informal caregivers of different ages in a cost-effective manner [4,5].

Childhood and adolescence are critical periods with unique developmental and health care needs [6]. Children and adolescents undergo dramatic changes in growth and development in physical, cognitive, and social domains. With brain development, maturation of their cognitive abilities allows higher levels of thinking, influencing their understanding of self and social surroundings [7]. The etiology of diseases varies depending on this developmental trajectory. Responses to disease differ based on social function such as language acquisition, which allows more mature self-functioning with different levels of autonomy depending on the developmental stage. In this process, family and peer influence are important [8].

Considering these critical changes, informal caregivers also have unique needs while caring for ill children and adolescents. Informal caregivers have a responsibility to optimize the healthy development of their children as part of the parenting process [9]. Knowledge of the physical and mental development of children with health care needs allows informal caregivers to evaluate disease processes along with normal developmental responses [10]. Optimal care can be provided by enhancing child self-care to maximize patient autonomy; this ultimately allows for better patient outcomes. Thus, parental understanding of the developmental stages and physical and psychosocial functioning of their children is vital [11]. Moreover, the responsibility of providing continuous intensive care can add extra burden and stress to informal caregivers [8]. Relationships with emotional attachments can also produce higher levels of stress and feelings of guilt [12].

Unique needs in the disease and caregiving trajectories may be met using the benefits of Internet-based health care service and resources. It is important to know how Internet-based health care services and resources have been used and what their perceived benefits and barriers are. To our knowledge, there have been no systematic reviews conducted to discriminate informal caregiver needs from those of their care recipients or from those caring for adults. Our integrative review on this topic proposed to synthesize the current understanding and state of the art regarding health-related Internet use by informal caregivers of children and adolescents with health care needs in order to identify better ways to help them. The aims of this integrative review were to (1) explore how Internet-based health care services and resources have been used by informal caregivers of children with health care needs; (2) identify the perceived benefits and barriers in health-related Internet use; and (3) examine the conceptual and methodological issues of the previous studies on this topic.

## Methods

This integrative review was based on a comprehensive approach of a literature search [13] and the Preferred Reporting Items for Systematic Reviews and Meta-Analyses guidelines [14].

### Search Strategy

For this integrative literature review, an initial literature search was conducted from July 2014 to September 2014 and an additional search was conducted in July 2015. The first search in 2014 did not specify the types of informal caregivers who took care of sick children. After we analyzed the first 14 studies chosen, it was found that most of informal caregivers related to this age group of care recipients were parents. The authors chose to conduct additional searches specifying parent(s) who are primarily responsible for child care.

Initially, we searched for studies published from 2009 to 2014, very few studies met this strict time period. Thus, we decided to expand the publication period to the years 2004 to 2014. A total of 6 computerized databases were searched: PubMed, the **Cochrane Library,** the Cumulative Index of Nursing and Allied Health Literature (CINAHL), PsycINFO, the Education Resources Information Center (ERIC), and EMBASE. Additional manual searching was performed on Google Scholar based on an ancestry search of citation and reference lists obtained from retrieved articles. Additional searching was also performed within the journals Pediatrics and the Journal of Medical Internet Research [15].

**Figure 1:**
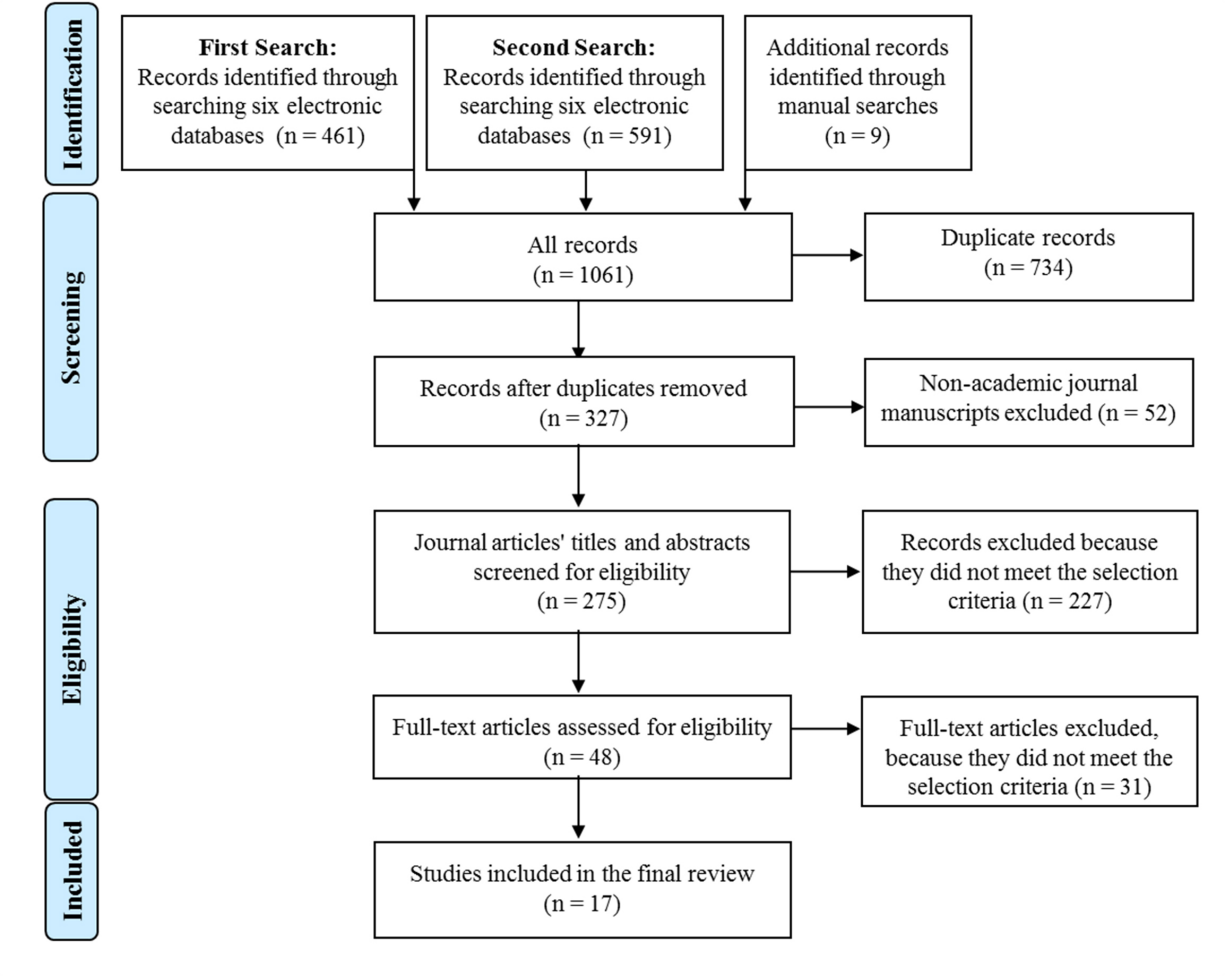
PRISMA flow diagram outlining the search and review process.

The initial set of search terms consisted of “health-related Internet use,” “eHealth,” “Internet use for health-related purpose(s),” “Web-based resource(s),” and “online resources,” combined with “caregiver” of “child,” “adolescent,” “student,” “youth,” and “teen.” For the second search, “caregiver” was replaced with “parent(s).” Titles, abstracts, and full texts were selected by applying the following inclusion and exclusion criteria. If the article was a systematic review, Cochrane review, literature review, or expert opinion, we used it as background information and examined its references but did not include it in the analysis. The first search results consisted of 470 records of which 14 studies were selected for the review. The second set of search results consisted of 591 records of which 3 studies were added for the review (see Figure 1).

### Eligibility Criteria

Inclusion criteria were as follows:

- Children with health care needs receiving informal care were limited to ages younger than 22 years because of discrepancies in legal age among different countries
- Primary study participants recognized themselves as informal caregivers (or parents) of children with health care needs
- Informal caregivers (or parents) were limited to ages 21 years or older
- Studies could include no interventional Internet use in order to examine phenomenological usage in a natural setting without investigator manipulation
- Studies were observational studies to examine user-initiated Internet use
- Studies were written in English or Korean

Exclusion criteria were as follows:

- Study participants were mixed with other populations aged 20 years or younger
- Care recipients were mixed with other age groups aged 22 years or older
- Ages of recipients or informal caregivers (or parents) were not specified or reported
- Study participants were trained or professional health care providers (eg, physicians, nurses, or medical or nursing students)
- Intervention modality was combined with other non–Web-based technologies (eg, telephone)
- Studies using the Internet as a modality for survey, recruitment, or searching for relevant literature only focused on quality assurance of specific websites
- Studies were grey literature including dissertations, conference proceedings, papers or abstracts, or editorials

### Data Extraction, Analysis, and Synthesis

One author (HK) initially evaluated titles and abstracts by applying potential eligibility criteria to exclude articles that did not investigate Internet use in informal caregivers (or parents) of children with health care needs. Two authors (HK and EP) fully reviewed the selected articles after developing definite eligible criterion and had a satisfying level of agreement over 95% regarding final selection of the articles. Two authors (HK and EP) entered data from selected articles into an analysis table, and an outside validator (AS) with a Master of Library and Information Management degree examined the articles and edited the table entries for accuracy (99% verification). To answer research questions 1 and 2, the coding scheme was developed based on our study purposes and Eysenbach’s framework [1]. To evaluate the quality of study methodologies responding to research question 3, we modified the guidelines of the Agency for Healthcare Research and Quality on rating the strength of scientific evidence considering our context [16].

## Results

### Characteristics of Study-Participating Care Recipients and Informal Caregivers

Of 17 studies, 7 were conducted in the United States. In the selected studies, children experienced a wide range of medical needs including (1) hearing loss [17]; congenital disease or developmental problems [18]; asthma [19,20]; hydrocephalus [21]; rare genetic diseases [22]; ear, nose, and throat surgeries [23]; and type 1 diabetes [24]. Study topics also included nonspecific diseases requiring primary health care [25-30], emergency care [30-32], and disability [33]. The majority of the 17 studies (12/17, 71%) used a wide range of age criteria even within a single study. Only 5 studies focused on specific age groups such as those aged 2-6 years [19,23,25,29] or preschoolers to 8th grade [19,23,24,29].

The selected studies had limitations in representing diverse populations including relationships to children, gender, race and ethnicity, insurance status, employment, education level, and the regions where informal caregivers live. The majority of studies were limited to parents or legal guardians (14/17, 82%); the remaining studies were of relatives as caregivers (3/17, 18%). The majority of participants were female, usually mothers [17,23,24,27-29,33]. A high proportion of racial and ethnic minorities were found in only 4 studies; African Americans were the largest group in these studies, with proportions ranging from 32% [21] to 83% [32]. Although the selected studies did not aim to recruit low-income families, 6 studies (35%) included low-income study participants (determined based on insurance and employment status). A high percentage of participants receiving Medicaid or government-provided insurance were included in 4 studies (56%-92%). More than 50% of study participants in the DeMartini and colleagues study [26] lived in a high-poverty area. In addition, 3 studies [17,29,33] reported a moderate to high proportion of those who were unemployed or with unsecured jobs. A summary of the study setting, study participants, and their characteristics is shown in Multimedia Appendix 1.

### Health-Related Internet Use

The definition, prevalence, purpose, and detailed types of general and health-related Internet use are summarized in Multimedia Appendix 2.

#### Prevalence of General Versus Health-Related Internet Use

General Internet use was defined based on access to the Internet via computer, cell phone, or other mobile handheld device [34]. The prevalence of general Internet use among informal caregivers was reported with a wide range, 62% to 99%. Half or more were daily users (49%-70%). In 2009, the generic search engines most frequently used were Google (79%), Yahoo (3%), and others (18%) [23]. Among general users, the most common places to access the Internet were at home (45%-87%) followed by anywhere using a smartphone (28%-71%), worksite (33%) and other places (3%-15%) including the library, community agencies, schools, and Internet cafés.

The prevalence of health-related Internet use varied (11%-90%) depending upon how it was operationalized and measured. Only one study used a comprehensive definition of health-related Internet use based strictly on Eysenbach's framework [1]—using the Internet for health-related information, support, and health care education [17]. The most common definition of health-related Internet users included people using the Internet for seeking health-related information for child caregiving [18,19,23,25,28,29,32]. Using a narrow definition was likely to be associated with a lower prevalence of health-related Internet use: 11% used the definition of those with access to care over the Internet focusing on email use [27], 58% used the definition of those with health-specific uses of digital technology [26], and 82% used a general definition of informal caregivers who used the Internet related to their children’s health [21,22,31,33].

#### Types of Health-Related Internet Use

##### Information (Content)

The most prevalent purpose for health-related Internet use was seeking information regarding child health care needs; 15% to 90% of caregivers knew how to find health-related information on behalf of care recipients [17-26,28-33]. In one study, many Internet users (87%) chose a generic search engine; almost half (44%) also visited specialized websites for specific health needs [17]. Only 35% used the Internet at the time of the care recipients’ diagnosis [18]; a small group of informal caregivers (9%) sought Web-based information immediately prior to their onsite clinic visit [31].

Informal caregivers were not confident in their ability to appraise health-related information found on the Internet or distinguish the quality of information and support from health care providers. According to Knapp and colleagues [28], only half of the users felt confident enough to evaluate the quality of Web-based information, although the Internet was the most commonly used source for health information according to Bouche [25]. From 10% to 50% of informal caregivers discussed the information found through Internet searches with their health care providers during onsite clinic visits [17,23]. Half of them stated that their health care providers were interested in the Web-based information [17].

##### Communication

Informal caregivers used the Internet for communicating with their health care providers or peers [21,27,30,32]. The informal caregivers expressed a strong interest in using the Internet and emails to communicate with primary health care providers (80%-86%) [31,33] and health care providers in the emergency department (93%), including receiving lab results [32]. In addition, informal caregivers thought that electronic communication between primary and emergency department care providers would be helpful (34%) [32]. The informal caregivers also expressed a strong interest in using the Internet and emails to contact organizations related to health concerns and promotion (36%) [31,33].

As a communication method with their health care providers, the informal caregivers wanted to receive information via an electronic newsletter about current disease trends (77%), discharge instructions (66.0%), and educational content about common illnesses (73%) [32]. These findings are similar to the study showing the information that respondents want to receive from their health care providers online includes common infections (77%), age-appropriate activities (73%), healthy eating (71%), required well-child visits and screening tests (65%), and resources in community (62%) [26,30].

##### Support (Community)

The Internet was also commonly used by informal caregivers for obtaining emotional and social support [17,21,22,24]. Almost 30% used emotional support groups and 35% used the Internet for communication with parents in similar situations [20]. As many care recipients had life-long chronic illnesses, the Internet played an important role in helping informal caregivers cope with their emotions by having more information [22,24]. Internet support groups helped informal caregivers adjust to their children’s condition [22]. Peer communication using email was beneficial for expanding their interaction beyond the membership of a certain online group [21].

##### Education

The Internet was also commonly used by informal caregivers to educate themselves about obtaining care for themselves and their care recipients simultaneously [17]. To educate themselves regarding their personal health care, 86% of informal caregivers found the Internet helpful in learning about diseases [21], and 78% of participants used YouTube for educational videos related to health. Most informal caregivers wanted guidance and recommendations from their health care providers about which online resources to use [21]. Only 58% of those who sought information regarding their personal health care questions trusted the information received, and then only sometimes or somewhat [21].

##### eCommerce

None of the studies investigated any purchases of medical products or medications via online shopping.

#### Associated Factors of Health-Related Internet Use

There was evidence that a higher education level in informal caregivers was associated with more frequent use of the Internet related to health [17,21,28], which is consistent with findings from previous studies [2,35]. Higher education levels seemed to be correlated with adequate health literacy [19,20]. A digital divide existed for racial and ethnic minorities such as African Americans and Hispanics and among non-English-speaking groups [21,27,28,32].

Caregiving-specific factors of health-related Internet use included (1) a strong intention to understand children's health information [29], (2) unmanageable situations beyond the capacity of parental adjustment [22,24], and (3) specific treatment requirements of the children [18,24]. However, no relationship with health-related Internet use was found regarding geographic location, age of parents, status of disease, or number of consultations with primary care providers [17,21,25].

#### Perceived Benefits

Informal caregivers stated that it was easy to find helpful information regardless of the time and their location. Information helped informal caregivers understand a child’s medical condition [18,31], understand specific treatment [19,23], and make decisions about treatment [23,26]. In terms of Internet use as a support system, they were highly satisfied with Internet-based parental support groups, citing obtaining usable ideas, improved informal caregiver relationships with their children, finding people to trust, and seeking stress-coping strategies as specific benefits [22].

#### Perceived Barriers

There were several barriers that informal caregivers encountered using the Internet for health-related purposes. The quality of websites was a main barrier [17]. Only half felt confident assessing the quality of Web-based information [28]. This may explain why 94% of participants responded that they were not able to find the information they wanted on the Internet [20,31]. Most participants did not remember the specific health-related websites they used [31]. In addition, they hesitated to discuss the Web-based information they found with their health care providers [17,20,23]. This may be based on warnings from their health care providers not to trust Internet-based health information. Additional reasons caregivers do not use Internet health-related information may include personal logistical barriers, fear, and mistrust of information on websites [26]. Other barriers included cost, limited access, lack of knowledge, lack of time, medical disabilities, vision problems, concern about the negative effects of computer use, lack of transportation, and a lack of child care [31,33].

### Conceptual and Methodological Evaluations

#### Issues of Conceptualization

Most studies did not clearly define health-related Internet use. Using or accessing the Internet to find health-related information was the common operational definition. However, researchers did not provide the rationales for why they defined health-related Internet use based on the access to use Internet [34]. Only 2 studies (2/17, 12%) used theoretical frameworks to explain why health-related Internet use was important during the caregiving trajectory. The frameworks used were the theory of planned behavior [29,36] and Antonovsky’s concepts of sense of coping and coherence [22,37]. Other frameworks were used to understand the parental factors and their decisions to use online health information regarding diagnosis and treatment [29,36]. These frameworks considered the Internet a resource for helping informal caregivers reestablish a sense of coherence after they experienced stressful events due to their child's illness [22,37].

#### Study Design

All 17 studies were cross-sectional. The most frequently used study designs were quantitative (13/17, 77%) and prospective (16/17, 94%). Descriptive (8/17, 47%), correlational (9/17, 53%), qualitative (2/17, 12%) [22,24] and mixed-methods design (1/17, 6%) were also used [17]. Quantitative studies tended to report prevalence and predictors of health-related Internet use, and qualitative or mixed-method studies investigated informal caregiver perceptions of helpful and harmful health-related Internet use. There was no longitudinal study found to imply causality. Walsh and colleagues used multiple observation time points [29]. All of them were 1-group studies without any comparison group. Almost half of the 17 studies used a single recruitment site. Others used multiple sites including caregiver databases [25,33], online recruitment [29], multiple clinical sites [17,21,26], and large-scale clinical trials [19].

#### Study Sample

Convenience sampling was the most common. Only 4 studies used more rigorous systematic sampling methods based on probability such as random selection [20,28,33] and stratified sampling [25]. Sample sizes ranged widely from 10 to 2371. Based on the selected study design, data analyses, and justification of sample sizes, 8 studies (8/14, 57%) measured quantitative data and had adequate sample sizes, while 6 studies (5/13, 43%) had excessively large sample sizes. Only 2 studies [22,24] used a qualitative study design, but they had very small sample sizes (n=10 and n=27), although saturation was achieved. Most of the study participants spoke English, limiting generalizability to non-English-speaking populations. Nonresponder bias due to low response rates was identified in 5 studies; 4 of them collected data once [18,25,27,28] with response rates between 49% and 76%. Walsh and colleagues [29] collected data at 2 observation times and reported a 48% response rate at follow-up after 2 months from baseline.

#### Data Collection and Analysis

Almost all studies used surveys; one conducted unstructured interviews individually or as part of a focus group [22,24]. Onsite surveys seemed to be preferred (10/17, 59%) followed by postal (3/17, 18%), telephone (2/17, 12%), and online modality (1/17, 6%) surveys. Porter and Edirippulige conducted an online survey [17], and Walsh and colleagues used online recruitment [29], which decreased generalizability of this study to non-Internet users [38].

The gold standard instruments regarding health-related Internet use were the Health Information National Trends Survey questionnaire [39] and the Pew Internet and American Life study of consumers’ use of the Internet for health care information questionnaire [34]. However, only 2 studies used or modified these questionnaires [19,32]; most studies used their own. These survey questionnaires were developed with a lack of or poorly described psychometrics. Thus, the validity and reliability of these investigator-developed instruments were not well established.

Most of the types of data analyses were descriptive: univariate analyses (chi-square, student *t*, Pearson *r*, or other nonparametric tests), descriptive frequency statistics (numbers, percentages, means, and standardized deviations), or multivariate analyses (multivariate analyses of variance, linear regressions, or logistic regressions). The types of analysis statistics were appropriately chosen based on levels of data and measurement types. However, there was very limited information about reporting statistical assumptions checked, handling missing data, reporting pre-analysis, or including significant covariates in the analysis. The summary of methodological evaluation is shown in Multimedia Appendix 3, and the evaluation criteria are explained in Multimedia Appendix 4.

## Discussion

### Principal Findings

This integrative literature review provides an important understanding of how informal caregivers of children with health care needs used Internet-based information and support systems. In spite of variability, health-related Internet use among informal caregivers of children is similar to that of caregivers of adults [2,40,41]. The most prevalent use of the Internet is for disease-specific information about disorders and treatments, affecting decision making about treatment. Social support for emotional needs via a virtual community was also commonly used by informal caregivers.

### Comparison With Prior Work

A digital divide exists for racial and ethnic minorities and those with low education and limited Internet access. Consistent with previous study findings, the predictive values of education levels were well represented [41]. A secondary data analysis using National Alliance for Caregiving data found that those with a college-level education were 3.4 times more likely to be health-related Internet users than those who were educated to the level of high school or less [2]. A higher education level may be associated with either a higher level of knowledge of health-related resources, better computer skills, or more eHealth literacy [35].

Information is the key driving force behind increasing health-related Internet use. This is consistent with Internet use among informal caregivers of adult populations [5,40-42]. Informal caregivers of children with health care needs require comprehensive and timely information for monitoring their child's condition (85%), performing therapeutic support (65%), managing medications or treatment regimens (64%), giving physical therapies (44%), preparing a special diet (40%), or arranging available services in the community (39%) [43]. Most of the study participants were parents who needed information to make a decision on behalf of their child. Informal caregivers managed uncertainty through information exchanging behavior [44]. Thus, health-related Internet use provided supplemental resources to ensure that informal caregivers knew how to deal with their children (84%), how to advocate for themselves (72%) or on behalf of the child (85%), and how to manage financial issues (63%) [43].

Support through online communication and community is the second driving force of health-related Internet use among informal caregivers of children with health care needs. Informal caregivers' emotional stress has been shown as a need variable that facilitates their use of resources [45]. The secondary analysis using National Alliance for Caregiving data found that the higher the emotional stress being experienced by dementia caregivers, the more health-related Internet use they reported [2]. Based on the stress-appraisal theory [46] and the stress process model [45], there is a positive relationship between recognized stress levels and efforts to alleviate stress. Thus, health-related Internet use may be considered a coping strategy for reducing informal caregiver subjective stress or burden [46] and a way to modulate between caregiving stress and negative outcomes [45].

### Implications for Current Practice and Research

Our study found that health-related Internet use is highly prevalent and that caregivers need better guidance identifying quality information sources. Our study assists clinicians and researchers who want to provide information and communication technology (ICT)-based interventions for improving the quality of care for informal caregivers and their care recipients. First, information should be evidence-based and written at a sixth grade level or lower to include informal caregivers with low levels of education [47]. Second, informal caregivers and their care recipients should be provided with educational opportunities to learn about computers, the Internet, and multimedia devices and technologies based on the consumer health informatics guidelines [48]. Third, Internet-based interventions should be consumer-centered reflecting their needs for health care, preferences, and capacity to use. Recent work by Davies and colleagues has provided a great example of this approach. This research project consisted of four steps: (1) a literature review to assess what is known about the selected topic, (2) the development of a health information website based on a standardized approach, (3) a usability study to reflect users’ lived experiences and opinions for further revision of ICT-based interventions, and (4) a feasibility study to examine the effect of the ICT-based intervention. Thus, we believe that this integrative literature review is a foundation for moving forward to develop consumer-centered Internet-based interventions for informal caregivers of children requiring special health care.

### Study Limitations

This study has several limitations. Although it adhered to the systematic review process, there might be potential errors and biases. Although clear inclusion and exclusion criteria were set up and a systematic review process was conducted, there is a possibility that reviewers might have missed appropriate studies in the search process. Multiple authors conducted the coding process independently and the results were compared, but potential biases of the authors might have influenced the review process. In addition, while two authors evaluated the quality of studies based on the guidelines of the US Department of Human Services Agency for Healthcare Research and Quality, there was still subjectivity in evaluating the studies.

Further research should overcome critical methodological limitations. First, the definition of health-related Internet use should be more clearly operationalized and stated. Consistent use of definitions and measures will allow us to compare prevalence across studies. Second, further studies should use probability sampling to increase generalizability of findings. Low response rates should be addressed to reduce self-selection bias. Third, there is an ongoing need to develop and use reliable and valid instruments to capture more comprehensive behaviors of health-related Internet use. Self-report bias is inevitable in survey studies, thus objective measures used for a long-term follow up would be helpful to conclude causality. Fourth, theory-based studies are required to explain the complexity of health-related Internet use. Last, more rigorous statistical analyses are required. For example, for studies recruiting participants from multiple sites, the heterogeneous characteristics of sites should be controlled as confounding variables. Further studies should consider institutional-level variables affecting characteristics of study participants from different sites.

### Conclusions

In spite of its limitations, this study provides important information for health care providers and policy makers to integrate the need of informal caregiver who take care of their children and adolescents when developing Internet-based interventions and services. There is sufficient evidence that health-related Internet use is highly prevalent, and there are increasing needs for better use of the Internet among informal caregivers. The findings of this review also reveal gaps in the literature, which could direct further research. In addition, the information provided in this study provides important implications in designing intervention programs for the target population.

## Appendix

Multimedia Appendix 1 Summary of characteristics of informal caregivers and care recipients from 17 studies.

Multimedia Appendix 2 Summary of prevalence and characteristics of health-related Internet use.

Multimedia Appendix 3 Methodological evaluation of study quality.

Multimedia Appendix 4 Modified guideline of Agency for Healthcare Research and Quality criteria.

## Abbreviations

CINAHL: Cumulative Index of Nursing and Allied Health Literature
ERIC: Education Resources Information Center
ICT: information and communication technology
