# Supplementary material for: Health-Related Internet Use by Informal Caregivers of Children and Adolescents: An Integrative Literature Review
Source: J Med Internet Res. 2016 Mar 3;18(3):e57. doi: 10.2196/jmir.4124 (PMC4796403; doi:10.2196/jmir.4124)
Supplement: Multimedia Appendix 1 [file jmir_v18i3e57_app1.pdf]

Table 1. Summary of characteristics of informal caregivers and care recipients from 17 studies

First author, Year	Country	Number of Participants	Characteristics of Children with Healthcare Needs		Characteristics of Their Informal Caregivers
			Age	Medical conditions	
Zuckerman, 2014 [30]	USA	458 (all completed)	Range: Birth - 12yrs	Receiving pediatric primary or urgent healthcare	• Parents<br>• Educated at least college=42%<br>• Non-Hispanic Caucasian=62%
DeMartini, 2013 [26]	USA	257 (all completed)	Median = 2.9yrs	Receiving pediatric primary healthcare	• Family to child<br>• Medicaid insured=92%<br>• Urban residents
Dudas, 2013 [27]	USA	300 (229 completed)	Range: Birth - 21yrs	Receiving pediatric primary healthcare	• Parents: Mothers=79%<br>• Caucasian=47%; AA=43%<br>• Medicaid insured=38%
Naftel, 2013 [21]	USA	300 (all completed)	• Range: 0 - 21yrs<br>• Mean=8	Shunted hydrocephalus	• Parents or legal guardian<br>• Caucasian=66%; AA=32%<br>• Medicaid insured=56%<br>• Rural residents=63%
Nordfeldt, 2013 [24]	Sweden	27 (all completed)	Range: 10-17	Type 1 Diabetes	• Parents: Mothers=63%
AlSaadi, 2012 [20]	Saudi Arabia	505	Range: Birth - 21yrs	Asthma	• Parents
Fagnano, 2012 [19]	USA	304 (all completed)	Range: 3 - 10yrs	Persistent asthma	• Family to child<br>• Urban residents
Saidinejad, 2012 [32]	USA	509 (all complete	Mean=8.23 (SD=4.07	Receiving healthcare in	• Family to child

		d)	)	the ED	• Caucasian=9%; AA=83%<br>• Medicaid insured=78%
Walsh, 2012 [29]	Australia	391(baseline) and 187 (follow-up)	Range: 0.5 - 10yrs	General health concerns	• Parents: Mothers=95%<br>• Not full-time employed=76%
Knapp, 2011 [28]	USA	4072 (2371 completed)	Range: 1 - 21yrs	Special healthcare needs	• Parents: Mothers=91%<br>• English speaker=77%
Gundersen, 2011 [22]	Norway	10 (all completed)	Range: 1.5 -16yrs	Rare genetic diseases	• Parents<br>• Educated at least high school=90%
Nogueira, 2009 [23]	Brazil	132 (all completed)	Range: 2 - 14yrs	Disease for ENT surgery	• Parents or legal guardian: Mothers=83%<br>• Educated at least diploma=73%
Bouche, 2008 [25]	France	2197 (1068 completed)	Range: 2 - 6yrs	Various conditions including allergy, asthma, preterm baby care, etc.	• Parents<br>• Single parent family=13%<br>• Educated at least high school=73%
Porter, 2007 [17]	Australia	207 (166 completed)	Range: Birth - 21yrs	Permanent hearing loss	• Parents or legal guardian: Mothers=89%<br>• Educated at least high school=85%<br>• Unemployed=36%
Goldman, 2006 [31]	Canada	1025 (950 completed)	Range: 1 - 21yrs	Receiving ED care in urban hospitals	Parents
Massin, 2006 [18]	Belgium	509 (389 completed)	Range: 3mos - 17yrs	Congenital heart disease	Parents

Blackburn, 2005 [33]	UK	788 (all completed)	Range: 0 - 17yrs	Disability	• Parents: Mothers=93%<br>• Caucasian=96%; AA=4%<br>• Unemployed=56%

Notes for abbreviations: USA=United States of America; UK=United Kingdom; Yrs=Years; Mos=Months; SD=Standard deviation; ED=Emergency department; ENT=Ear, nose, and throat; AA=African American
