# Supplementary material for: Health-Related Internet Use by Informal Caregivers of Children and Adolescents: An Integrative Literature Review
Source: J Med Internet Res. 2016 Mar 3;18(3):e57. doi: 10.2196/jmir.4124 (PMC4796403; doi:10.2196/jmir.4124)
Supplement: Multimedia Appendix 2 [file jmir_v18i3e57_app2.pdf]

Table 2. Summary of prevalence and characteristics of health-related Internet use						
First author, Year	General Internet use		General health-related Internet use		Within health-related Internet use group	
	Prevalence	Characteristics	Definition	Prevalence	Purpose	Characteristics
Zuckerman, 2014 [30]	73%	• 87.3% accessed at home<br>• 73% =Daily email users	Caregivers who obtained health information online	47-78%	• Information (78%)<br>• Communication (47%)	Facilitator of information use: Higher parent educational attainment<br>Facilitators of communication with a healthcare provider: Higher parent age, higher parent educational attainment,<br>English household language, and daily email use
DeMartini, 2013 [26]	97%	• 80% accessed at home<br>• 71% accessed using phone<br>• 91%=Email users<br>• 78%=Facebook users<br>• 55%=Daily users	Caregivers' health-specific uses of digital technology	58%	Information	• 15%=Daily user and 58%=weekly user<br>• 6%=Specific search for hospital information<br>• 71%=Used online information approved by medical providers<br>• 53%=Used online information for decision makings about their child's health<br>• Preferred contents: Common infections (77%), developmental activities (73%), healthy eating (71%), regular check-up(65%),

						&regional resources (62%)
Dudas, 2013 [27]	76%	Email users are more likely to have a college or greater education, higher income, and private insurance.	Caregivers' access to care over the Internet focusing on email use	11%	Communication (74%)	• 86%=Wanted to email to their doctors<br>• 79%=Agreed that more doctors should offer email communication<br>• 40% wanted to receive lab results via email.<br>• Disparities of race (AA)
Naftel, 2013 [21]	92%	• 63%= Daily user<br>• 60%=Social media user<br>• Place of access to Internet: Home and smart phone	e-Caregivers who use the Internet related to their children's health	82%	• Information (82%)<br>• Education (78%)<br>• Support group (29%)<br>•Communication (35%)	• Among e-caregivers:<br>- 86%=Helpful in learning about disease<br>- 90%=Interested in doctors' guidance<br>in use of online resources<br>- 58%=Sometimes trusted information<br>- 95%=Accessed social media<br>- 78%=Used educational videos (YouTube)<br>• Disparities of race (AA), low income, and lower education<br>• No association related to geographic location, age of parents, and status of disease.
Nordfeldt, 2013 [24]	96%	• 85% at home	Caregivers who obtained	52%	• Information<br>• Management of stressful life	Unspecified Internet sources used for support and information related

			health information and support		event	to type 1 diabetes. The use increases trust and suitability of the disease and finally meet parental emotional needs.
AlSaadi, 2012 [20]	-	-	Caregivers who obtained health information	79%	• Information	• Reasons for internet use were reassurance, clarification, online consulting, and new management schemes.<br>• Facilitator of information use: Higher parent educational attainment
Fagnano, 2012 [19]	73%	• 45% at home<br>• 54%=Email account	Caregivers who obtained health information online	52% - 62%	Information	• 90%=Online information was easy to read<br>• 85%=Online information was easy to understand<br>• Facilitator: Adequate health literacy
Saidinejad, 2012 [32]	99%	• 62% at home<br>• 70%=Daily users<br>• 96%=Have an email account<br>• 60%=Facebook account	Caregivers interested in receiving health care-related information via electronic means	_	• Information<br>• Communication<br>• Education	• 93%=Interested in electronic communication from the ED<br>• 34%=Interested in electronic communication channel with primary care providers<br>• 77%=Interested in receiving ED newsletter<br>• 66%=Discharge instruction<br>• 73%=Education about

						common illness<br>• Disparities of ethnicity (Hispanic)
Walsh, 2012 [29]	_	Mean hours to use Internet per week=26.32 hours	Caregivers using online health information for their child’s health care	_	Information	Facilitator: Strong intention to understand child health information
Knapp, 2011 [28]	82%	49%=Daily user	Caregivers knowing how to find health-related information via online for their children	74%	Information	• 50%=Felt confident in finding high quality information online<br>• Disparities of race (AA) and non-English speaking populations
Gundersen, 2011 [22]	_	_	Caregivers using Internet to help their children	_	• Information<br>• Coping with their emotions<br>• Management of stressful life event<br>• Reestablishment of sense of coherence	Facilitator: Unmanageable situations beyond parental adjustment
Nogueira, 2009 [23]	98%	Common generic search engines=Google (79%), others (18%),	Caregivers using Internet for health-related	90%	Information	10%=Discussed the web-based information with their physician during onsite clinic visit

		and Yahoo (3%)	information			
Bouche, 2008 [25]	–	–	Caregivers using Internet for health-related information	52%	Information	• Internet is the most commonly used health information source.<br>• No association between health-related Internet use and the number of consultations with primary care providers.
Porter, 2007 [17]	67%	67% =Comfortable using the Internet	Caregivers using Internet for health-related information, support, and healthcare education	44 - 87%	• Information (87%)<br>• Emotional and social support (55%)<br>• Education (44%)	• 87%=Used a generic search engine<br>• 44%=Used specialized medical websites<br>• 11%=Used websites recommended by family and friends<br>• 52%=Discussed the web-based information with their physician during onsite clinic visit<br>• 58%=Their doctors were interested in the web-based information<br>• Facilitator: Higher levels of education<br>•No age effect on health-related Internet use
Goldman, 2006 [31]	87%	• 15% in a library, Internet café, or other public place<br>• 75%=Email user	Caregivers using Internet for their children's health conditions	56%	Information	• 8.5%=health-related Internet use before onsite clinic visits<br>• Few remembered the specific websites informal caregivers used.

		• 60%=Daily email user				• 80% of parents used and wanted to receive results electronically.<br>• 95%=Internet was very helpful in understanding their child's illness<br>• 94%=Cannot find online information
Massin, 2006 [18]	62%	• 74% at home<br>• 33% at work<br>• 4% at school<br>• 3% at cyber cafe	Caregivers using Internet for health-related informatio n	15% - 35%	Information	• 35%=health-related Internet use at the time of diagnosis<br>• Facilitator: Care-recipient's specific treatment need
Blackburn, 2005 [33]	75%	• 83% at home<br>• 28% access using phone	Caregivers using Internet related to caring	72%	• Information (72%)<br>• Contact with organizations related to health (36%)	• Technical problems:<br>- Longer time to find the information (86%)<br>- Unsuccessful searches (85%)<br>- Websites were difficult to navigate (79%)<br>- Too slow Internet connection (73%)<br>- Difficult to understand (60%)<br>- Difficult to use software (57%)<br>• Circumstantial barriers:<br>- Lack of time due to demands of care (57%) or due to other commitments (61%)

						- Cost of internet (22%)<br><br>- Costs of equipment (15%)<br><br>- Fear about technology (13%)
Notes for Abbreviations: ED=Emergent department; AA=African American						
