# Supplementary material for: Health-Related Internet Use by Informal Caregivers of Children and Adolescents: An Integrative Literature Review
Source: J Med Internet Res. 2016 Mar 3;18(3):e57. doi: 10.2196/jmir.4124 (PMC4796403; doi:10.2196/jmir.4124)
Supplement: Multimedia Appendix 3 [file jmir_v18i3e57_app3.pdf]

Table 3. Methodological Evaluation of Study Quality

First Author, Year	Research design	Conceptual framework	Sampling	Data collection method and instrument	Analysis method	Threats to validity
Zuckerman, 2014 [30]	Quantitative; Cross-sectional; Correlational; Prospective	○ None	● Convenience sampling in a single site	○ Onsite survey using investigator-developed questionnaires	● Descriptive frequency statistics; logistic regression	● Self-report and self-selection bias; limited generalizability for non-English speakers
DeMartini, 2013 [26]	Quantitative; Cross-sectional; Descriptive; Prospective	○ None	● Convenience sampling from multiple sites	◐ Onsite survey using investigator-developed questionnaires and 4-point Likert scale of digital technology	● Descriptive frequency statistics; Univariate analysis (Chi-square test)	● Self-report and self-selection bias; heterogeneity between two sites
Dudas, 2013 [27]	Quantitative; Cross-sectional; Correlational; Prospective	○ None	● Convenience sampling in a single site	○ Onsite survey using investigator-developed questionnaires	● Descriptive frequency statistics; logistic regression	● Self-report and self-selection bias; questionable reliability between self- and clinician-report; limited generalizability for non-English speaking
Naftel, 2013 [21]	Quantitative; Cross-sectional; Descriptive; Prospective	○ None	● Convenience sampling from multiple sites	○ Onsite survey using investigator-developed questionnaires	● Descriptive frequency statistics; Univariate analyses (Chi-	● Self-report and self-selection bias; limited generalizability for non-English speakers

					square & t tests)	
Nordfeldt, 2013 [24]	Qualitative; Cross-sectional; Descriptive; Prospective	○ None	● Purposive sampling based on the child age and gender	Structured focus group discussion	● Inductive qualitative content analysis	● Self-report and self-selection bias
AlSaadi, 2012 [20]	Quantitative; Cross-sectional; Correlational; Prospective	○ None	● Random sampling from multiple sites	○ Onsite survey using investigator-developed questionnaires	● Descriptive frequency statistics; logistic regression	● Self-report and self-selection bias; limited generalizability outside of Saudi Arabian
Fagnano, 2012 [19]	Quantitative; Cross-sectional; Correlational; Prospective	○ None	● Convenience sampling from a clinical trial	● Onsite survey using a modified Health Information National Trends Survey questionnaire [35]	● Descriptive frequency statistics; logistic regression	● Self-report and self-selection bias; limited generalizability due to limited recruitment method
Saidinejad, 2012 [32]	Quantitative; Cross-sectional; Correlational; Prospective	○ None	● Convenience sampling in a single site	● Onsite survey using a modified Pew and the American Life study of consumers' use of the Internet for healthcare information questionnaire[31]	● Descriptive frequency statistics; univariate analyses (Chi-square & t tests); logistic regression	● Self-report and self-selection bias; limited generalizability for non-English speakers; overpowering with large sample size (type 1 error)
Walsh, 2012 [29]	Quantitative; Cross-sectional with multiple measures; Correlation	● Theory of planned behavior	● Convenience sampling through online approach	● Telephone survey using investigator-developed questionnai	● Descriptive frequency statistics; univariate analysis	● Self-report and self-selection bias; high drop-out rate (52%); limited

	al; Prospective			res (psychometric information reported)	(Pearson r test), MANOVA; hierarchical linear regression	generalizability for non-Internet users
Knapp, 2011 [28]	Quantitative; Cross-sectional; Correlational; Prospective	○ None	● Random sampling in a medical services network program	◐ Telephone survey using investigator-developed questionnaires and eHealth Literacy Scale	● Descriptive frequency statistics; logistic regression	● Self-report and self-selection bias; low response rate (58%); overpowering with large sample size (type 1 error)
Gundersen, 2011 [22]	Qualitative; Cross-sectional; Descriptive; Prospective	● Antonovsky's concepts of sense of coping and coherence	● Convenience and purposive sampling	◐ Unstructured in-depth interview	● Content analysis	○ Self-report and self-selection bias; questionable validity and reliability from one rater and coder
Nogueira, 2009 [23]	Quantitative; Cross-sectional; Descriptive; Prospective	○ None	● Convenience sampling in a single site	○ Onsite survey using investigator-developed questionnaires	◐ Descriptive frequency statistics	● Self-report and self-selection bias; limited generalizability for non-English speakers
Bouche, 2008 [25]	Quantitative; Cross-sectional; Correlational; Prospective	○ None	● Stratified sampling(type and location) from multiple sites	○ Postal survey using investigator-developed questionnaires	● Descriptive frequency statistics; logistic regression	● Self-report bias; low response rate (49%)
Porter, 2007 [17]	Mixed methods; Cross-sectional; Descriptive	○ None	● Convenience sampling from multiple	◐ Online survey using investigator	● Descriptive frequency statistics;	● Self-report and self-selection bias; unclear eligibility of

	; Prospective		sites	-developed questionnaires (reliability tested)	univariate analysis (Chi-square test); content analysis	study participants; limited generalizability for non-Internet users and non-English speakers; heterogeneous study sites
Goldman, 2006 [31]	Quantitative; Cross-sectional; Descriptive; Prospective	○ None	● Convenience sampling in a single site (stratified by visit day)	◐ Onsite survey using investigator-developed questionnaires (reliability tested)	● Descriptive frequency statistics; univariate analyses (Chi-square & t test)	○ Self-report and self-selection bias; overpowering with large sample size (type 1 error); heterogeneous study participants; limited generalizability for non-English speakers
Massin, 2006 [18]	Quantitative; Cross-sectional; Correlational; Prospective	○ None	● Convenience sampling in a single site	○ Postal survey using investigator-developed questionnaires	◐ Descriptive frequency statistics; univariate analysis (Chi-square test); logistic regression	○ Self-report and self-selection bias; overpowering with large sample size (type 1 error)
Blackburn, 2005 [33]	Quantitative; Cross-sectional; Descriptive; Prospective	○ None	● Systematic sampling from the Carers UK database	◐ Postal survey using investigator-developed questionnai	● Descriptive frequency statistics; univariate analysis	● Self-report bias; low response rate (40%); heterogeneous primary data sources;

	res (reliability tested)	(Chi-square test)	overpowering with large sample size (type 1 error)

Notes: Based on the guidelines of the Agency for Healthcare Research and Quality criteria [16]; ●=Fully met the criteria; ◐=Partially met the criteria; and ○=Not met the criteria.
