# Supplementary material for: Health-Related Internet Use by Informal Caregivers of Children and Adolescents: An Integrative Literature Review
Source: J Med Internet Res. 2016 Mar 3;18(3):e57. doi: 10.2196/jmir.4124 (PMC4796403; doi:10.2196/jmir.4124)
Supplement: Multimedia Appendix 4 [file jmir_v18i3e57_app4.pdf]

Table 4. Modified Guideline of Agency for Healthcare Research and Quality Criteria	
	Criteria
Conceptual framework	• Appropriate use of conceptual framework<br>Clear definition of concept(s)
Sampling	• Description of study populations<br>• Sample size justification<br>• Specific inclusion/ exclusion criteria for all groups<br>Study groups comparable to non-participants with regard to confounding factors
Data collection method and instrument	• Outcome measures of precision<br>Measurement method standard, valid, and reliable
Data analysis	• Appropriateness of statistical models<br>• Adequacy of description and reporting of statistical analysis<br>Extent to which all analysis that should have been done were done
Threats to validity	• Threats to internal validity – Degree to which the study provides valid evidence for the population and setting<br>• Threats to external validity – Extent to which the evidence is generalizable to the population
